# Parity Property of Hexagonal Sliding Puzzles

**DOI:** 10.1007/s44007-025-00160-2

**Published:** 2025-05-07

**Authors:** Manuel Estévez, Ray Karpman, Érika Roldán

**Affiliations:** 1 ScaDS.AI, Leipzig University, Leipzig, Germany; 2 Department of Mathematics, Otterbein University, Columbus, OH USA; 3 Max Planck Institute for Mathematics in the Sciences, Leipzig, Germany

**Keywords:** The 15 Puzzle, Sliding puzzles, Configuration spaces, Even permutation, Alternating group, God’s number, Solvability of puzzles, Breadth-first search, 00A08, 05A20, 05C25, 05A05, 05B45, 05B50, 05D9

## Abstract

We study the puzzle graphs of hexagonal sliding puzzles of various shapes, and with various numbers of holes. The puzzle graph is a combinatorial model which captures the solvability and the complexity of sequential mechanical puzzles. Questions relating to the puzzle graph have been previously studied and resolved for the 15 Puzzle, which is the most famous—and unsolvable—square sliding puzzle of all time. It is known that for square puzzles such as the 15 Puzzle, solvability depends on a parity property that splits the puzzle graph into two components. In the case of hexagonal sliding puzzles, we get more interesting parity properties that depend on the shape of the boards and on the missing tiles or holes on the board. We show that for large-enough hexagonal, triangular, or parallelogram-shaped boards with hexagonal tiles, all puzzles with three or more holes are solvable. For puzzles with two or more holes, we give a solvability criterion involving both a parity property, and the placement of tiles in *tight corners* of the board. The puzzle graph is a discrete model for the configuration space of hard tiles (hexagons or squares) moving on different tessellation-based domains. Understanding the combinatorics of the puzzle graph could lead to understanding some aspects of the topology of these configuration spaces.

## Introduction

Sliding puzzles are sequential mechanical puzzles which are solved by sliding certain pieces on a fixed board from a starting configuration to a target final configuration. We focus here on sliding puzzles whose boards consist of a finite subset of tiles of the square or hexagonal regular tessellations of the plane. If the board is completely covered by tiles, which are square or hexagonal depending on the selected tessellation, then the tiles can’t move at all. In this case, any sliding puzzle defined on that board is clearly unsolvable. If some tiles on the board are removed, then holes are created and it becomes possible to slide some of the tiles; as a consequence, some sliding puzzles become solvable. Here, we are interested in determining which sliding puzzles on a given board are solvable for boards of various shapes and with various numbers of holes. This problem has previously been solved for a large family of square sliding puzzles [8, 11, 12]. However, the answers for boards with hexagonal tiles were previously unknown.

The majority of sliding puzzles that have been physically created and commercialized have rectangular shaped boards and *n* square tiles usually labeled with integers going from 1 to *n*. However, there also exist more general *sliding block puzzles* where the pieces are unlabeled, or where the pieces consist of other very simple small polyominoes such as dominoes, triominoes, or tetrominoes. Edward Hordern had the biggest collection of sliding block puzzles in the world, which is now part of the Puzzle Museum’s collection [10]. In 1986 [4], Hordern wrote a wonderful book describing the more than 250 sliding block puzzles in his collection.

With very few exceptions, for sliding puzzles with labeled unit square tiles, if a board has exactly one removed tile, then a parity property determines if the puzzle is solvable or not; with two or more holes, all sliding puzzles defined on the board are solvable. This was proved for the first time during the 1880s [8, 11], when mathematicians and the general public got attracted, obsessed, and even occasionally traumatized, by the most famous (unsolvable) sliding puzzle of all time: The 15 Puzzle [7].

**Fig. 1:**
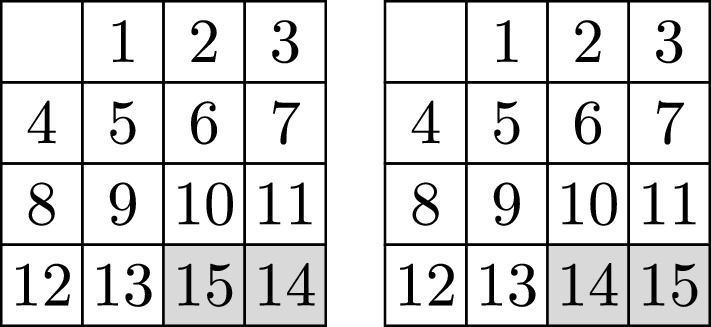
The most famous sliding puzzle is the 15 Puzzle which with our notation is represented by $R_{\square }(1;4)$. It is not possible to transform the puzzle on the left to the one on the right by sliding the tiles

The 15 Puzzle consists of a $4 \times 4$ square board divided into 16 square units with 15 square tiles labeled from 1 to 15 placed on the board and one empty square. The initial configuration of the tiles in the 15 Puzzle has the tiles from 1 to 13 placed in ascendant order (from left to right and top to bottom), and the 14 and 15 tiles interchanged. The target configuration of this puzzle leaves the first 13 ordered tiles fixed and interchanges the 14 and 15 tiles, so that all tiles are now arranged in ascendant order —see Fig. 1.

In 1879 [8], W. Johnson proved, using a parity argument, that the 15 Puzzle has no solution. He showed that if one starts from a specific configuration, it is impossible to reach any other configuration that is obtained by applying an *odd permutation* to the labels of the tiles. Recall that a permutation is *even* if it can be written as a product of an even number of transpositions–that is, of permutations which simply interchange two labels. A permutation is *odd* if it can be written as a product of an odd number of transpositions. Since every permutation is a product of transpositions, every permutation is therefore either even or odd.

Johnson’s proof works on a board in the shape of an $m_1 \times m_2$ rectangle with $m_1,m_2 \ge 2$ that has exactly one missing tile and where the rest of the tiles are unit squares labeled with consecutive integers. We say that a board (with the respective holes) has the *weak parity property* if we cannot reach any configuration corresponding to an odd permutation of the tiles by performing permitted slides. Hence, any rectangular board with one tile removed has the weak parity property.

Complementing Johnson’s analysis, W.E. Story proved in the second part of the same paper [11] that when starting from a fixed configuration, *all* configurations that can be obtained by an even permutation of the tiles will lead to a puzzle that has a solution. We say a board (with the respective holes) has the *strong parity property* if we are able to obtain all configurations that require an even number of transpositions of tiles from a fixed starting configuration.

Putting together the results by Johnson and Story tells us that it is possible to solve a sliding puzzle on a rectangular-shaped board with square tiles and exactly one tile missing, if and only if the following holds: if we slide the tiles of the starting configuration so that the missing tile is in the same position as in the target, the permutations of tiles in the starting and the final configurations have the same parity. In other words, Johnson and Story proved that the set of all configurations is partitioned into two big sets that are determined by parity, and that this partition characterizes when a puzzle has a solution (this means going from fixed initial to final configurations by sliding tiles on the board) or not. A further natural question then arises: how many more tiles need to be removed from the board for all of the sliding puzzles defined on it to be solvable? Trivially, for a rectangular-shaped board with unit square tiles, it suffices to remove two tiles. We call a board with the respective holes needed to have this property one that is *maximally connected*.

**Fig. 2:**
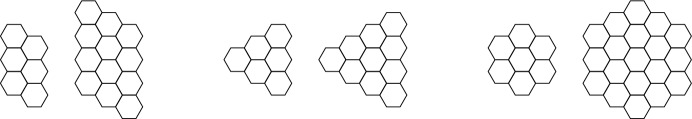
Left to right: A $2 \times 3$ parallelogram, a $3 \times 4$ parallelogram, a $3 \times 3$ triangle, a $4 \times 4$ triangle, a flower with 2 layers, and a flower with 3 layers

In this paper, we analyze the weak and strong parity properties and maximal connectivity of boards with different shapes and hexagonal tiles—see Fig. 2 for some examples of these boards. Of the families of hexagonal boards that we study here, only the family of parallelogram-shaped boards with hexagonal tiles has previously been defined and studied (by H. Alpert, [1]). In her paper, Alpert analyzes, with respect to the size of the boards, how quickly these parallelogram-shaped hexagonal sliding puzzles can be solved asymptotically. For this, she first proves that these parallelogram boards are maximally connected for sizes bigger than $5 \times 5$, with 6 or more hexagonal tiles removed and at least two of the removed hexagonal tiles sharing an edge. Thus, Alpert studies only maximally-connected boards with hexagonal tiles. With respect to this particular family of boards, we prove here that maximal connectivity is reached with three or more hexagonal holes (even for small parallelogram boards), and that they have the weak parity property (but not the strong parity property) with two holes. Thus, we show that, unlike the 15 Puzzle or more general square puzzles, the parallelogram sliding puzzles never have the strong parity property.

**Fig. 3:**
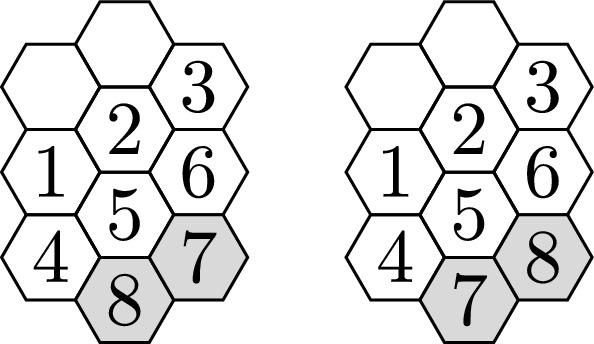
We prove that is not possible to transform the puzzle on the left into the puzzle on the right by sliding tiles

Given a specific sliding puzzle on a board, we can have a sense of how difficult it will be to solve it by knowing the minimum number of single slides required to get from the starting to the targeted final configuration of the puzzle. This defines a distance on the set of configurations and a natural way of measuring the complexity of a sliding puzzle. In Fig. 3, we show an example of a hexagonal sliding puzzle with no solution. With this perspective, the *most difficult puzzles* that can be defined on a given board are those that require the most slides to be solved. This is captured by the *God’s number*, which is the maximum of the distances between configurations.

In the specific case of the board of the 15 Puzzle, its God’s number has been computed and it is equal to 80 [2]. A well-known open problem is to determine the God’s number for bigger square-shaped (or rectangular-shaped) boards. The problem of finding the God’s number of square-shaped boards with labeled square tiles is NP-Hard [3]. In Sect. 5 we also give bounds for the God’s number of some hexagonal sliding puzzles. In the rest of this section, we give precise statements of our main results.

### Main Results

#### [Style1 Style2]Definition 1

Let *B*(*h*) denote the *board* *B* with *h* holes; that is, with *h* unoccupied positions of the board. We define the *puzzle graph* of *B*(*h*), denoted by *puz*[*B*(*h*)], as the graph whose vertices $C_i$ are each one of the possible configurations of the labeled tiles on the board *B* and that has an edge between two vertices $C_i$ and $C_j$ whenever it is possible to go from configuration $C_i$ to configuration $C_j$ by sliding one tile into a hole.

#### [Style1 Style2]Definition 2

The *permutation group with* *n tiles* of *B*(*h*) is the symmetric group $S_n$. An element $\sigma \in S_n$ induces an action on a configuration *C* by permuting the tile labels with the *h* holes in fixed positions. We denote the action of $\sigma \in S_n$ on *C* by $\sigma \cdot C$, which is again some configuration of *B*(*h*). We denote the subgroup of $S_n$ consisting of even permutations, known as the alternating group by $A_n$.

**Fig. 4:**
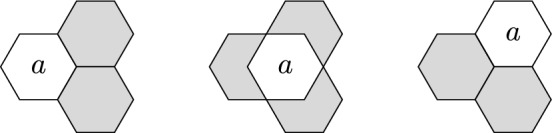
As noticed in [1], a hexagonal tile that is adjacent to a pair of holes may slide into either neighboring hole

If two tiles are removed and share an edge, topologically both together would count as only one hole. Nevertheless, we count each unoccupied position of the board as a distinct hole. When the number of holes does not need to be specified, we will abuse notation and denote the board with any number of holes as *B*. In some cases, it is useful to label the holes on a puzzle board just as we do with the tiles. With this holes labeled, we can view the action of sliding a labeled tile *a* into a labeled hole e.g. 0 as equivalent to interchanging the positions of the tile *a* and the hole 0.

#### [Style1 Style2]Definition 3

The *permutation group with n tiles and h holes* *B*(*h*) is the symmetric group $S_{n+h}$. An element $\tau \in S_{n+h}$ acts on a configuration *C* by permuting the tile and hole labels, and the action of $\tau \in S_{n+h}$ on *C* is denoted by $\tau \cdot C$, which is again some configuration of *B*(*h*).

#### Remark 1

Every slide on the tiles of a board *B*(*h*) is an element $\tau \in S_{n+h}$, but an arbitrary element $\tau ' \in S_{n+h}$ may not be a slide. We will refer to a slide of the tiles by the letter $\tau$. On the other hand, when referring to some permutation of the *n* tile labels with fixed hole positions we will use the letter $\sigma$.

#### [Style1 Style2]Definition 4

We say a configuration of a board *B* is *isolated* if no slides are possible. A configuration is *non-isolated* if there is at least one tile that can slide.

For a board with square tiles, isolated configurations are only possible for boards with no holes, as it is always possible to slide a square tile into a hole that shares an edge with the tile. For a board with hexagonal tiles, a tile can slide to a neighboring empty hexagonal hole if and only if there are two adjacent empty hexagons that both share an edge with the tile that will move—see Fig. 4.

#### [Style1 Style2]Definition 5

We say that *puz*[*B*(*h*)] has the *weak parity property* if whenever two non-isolated configurations $C_i, C_j$ with *n* the holes in the same positions are in the same connected component, then there exists an even permutation $\sigma \in A_n \trianglelefteq S_n$ such that $\sigma \cdot C_i = C_j$.

#### [Style1 Style2]Definition 6

We say that *puz*[*B*(*h*)] has the *strong parity property* if it has the weak parity property and its puzzle graph has exactly two components, let’s say $\mathcal {C}^1$ and $\mathcal {C}^2$, that contain all (non-isolated) configurations.

Hence, *puz*[*B*(*h*)] has the strong parity property if two non-isolated configurations $C_1,C_2$ of *B*(*h*) are in the same connected component of *puz*[*B*(*h*)] exactly when there is an even permutation $\sigma \in A_n$ such that $\sigma \cdot C_1 = C_2$. In other words, the alternating group $A_n$ acts transitively in each of the two components $\mathcal {C}^1$ and $\mathcal {C}^2$.

#### [Style1 Style2]Definition 7

We say the puzzle graph of *B* is *maximally connected* if it has one large connected component containing all non-isolated configurations. We call the minimum number *h* such that the puzzle graph of *B*(*h*) is maximally connected the *connection number* of *puz*[*B*(*h*)].

We will denote the $m_1 \times m_2$ square grid board as $R_{\square }(h;m_1,m_2)$, where *h* represents the number of holes. In the particular case when $m=m_1=m_2$, we simplify the notation by $R_{\square }(h;m)$. Similarly, we will denote the $m_1 \times m_2$ parallelogram board with hexagonal tiles as , where *h* represents the number of holes. In the particular case when $m=m_1=m_2$, we simplify the notation by .

We investigate parallelogram boards of all sizes and with varying numbers of holes. We note that the $1 \times m_2$ square board $P_{\square }(1;1,m_2)$ has $(m_2-1)!$ connected components, one for each possible permutation of $m_2-1$ tiles, as we can only slide the tiles back and forth without changing their order. It is natural to ask if there exists a board shape for hexagonal sliding puzzles that exhibits analogous behaviour. We find that this is the case for  boards.

#### Theorem 1.1

For $m_1 =2$ and $m_2 \ge 2$,  has $(m_1m_2-h)!$ connected components containing non-isolated configurations.

For a rectangular-shaped board in the square case, the puzzle graph gets maximally connected when there are two holes. This is not the case for hexagonal sliding puzzles. For instance, we find that the $3 \times 3$ parallelogram with two holes exhibits a parity property similar to that seen in the 15 puzzle. If we remove three holes instead of two, the resulting puzzle graph is maximally connected, meaning that every sliding puzzle on a $3 \times 3$ board with three holes is solvable.

As previously mentioned, the only family of hexagonal boards that has been introduced and studied before are large parallelogram boards  by Alpert in 2020 [1].

#### Proposition 2

(Alpert [1]) For $m \ge 5$ and $h \ge 6$,  is maximally connected.

Alpert’s result states that if there are at least six removed tiles from a parallelogram hexagonal board that is at least of size $5\times 5$, then the puzzle graph of the board gets as connected as possible. Our next theorem improves on Alpert’s result by showing that we can reach maximal connectivity with only three holes. In fact, we shall see below that this is the exact number of tiles that need to be removed to reach maximal connectivity.

#### Theorem 1.2

For $m_1 \ge 3$ and $m_2 \ge 3$, the puzzle graph  is maximally connected.

Using this theorem, and an inductive approach involving *patching* together smaller boards, we explore parity properties, maximal connectivity, and the connection number of puzzle graphs for families of boards with other shapes. We now define a notation to use for these specific families of boards with hexagonal tiles.

Recall that we use  to denote a board in the shape of an $m_1 \times m_2$ parallelogram with *h* holes. Here, $m_1$ denotes the number of columns and $m_2$ the number of rows. When $m_1=m_2$, we simplify this notation to . When we wish to refer only to the shape of the board, not the number of holes, we drop the *h* and write .

Similarly, we write  to denote a board in the shape of an equilateral triangle with *m* tiles on a side. We write  to denote a board in the shape of a hexagon, again with *m* tiles on a side. To avoid confusion with the hexagonal shape of an individual tile, we refer to these hexagon-shaped boards as “flowers” in the text. We may think of a flower as being constructed by concentric rings of tiles around a central tile, hence  may be referred to as a flower with *m* layers.

#### Corollary 1.1

For $h \ge 3$, the puzzle graph of  is maximally connected for $m \ge 5$, and the puzzle graph of  is maximally connected for $m \ge 3$.

We now investigate boards with exactly two holes missing. Here, as in the case of rectangular boards with one hole, parity is a key consideration.

#### Theorem 1.3

The board  has the weak parity property for $m_1, m_2 \ge 3.$

#### Corollary 1.2

Any board with exactly two holes that is a sub-board of a parallelogram board has the weak parity property. Hence, boards of any shape that we explore in this paper with exactly two holes have the weak parity property.

#### Theorem 1.4

The connection number is 3 for the following boards:  with $m_1,m_2 \ge 3$,  with $m \ge 5$, and  for $m \ge 3.$

**Fig. 5:**
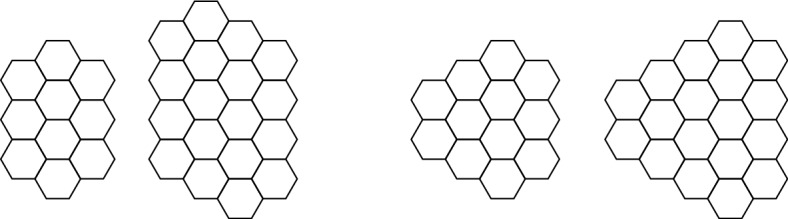
Left to right: The trimmed parallelograms  and , and the trimmed triangles  and

It might be natural to hope that, e.g., parallelogram-shaped boards have the strong parity property as well. However, this is not the case, essentially because tiles get “stuck” in corners of the board. To get around this problem, we define a *tight corner* of a board to be any tile with exactly two neighbors. We define a *trimmed board* as the result of removing all tight corners from an initial board. In principle, removing tight corners from a board could create new corners by removing neighbors of the tiles left behind. Fortunately, if we start with a large-enough triangle or parallelogram, the resulting trimmed board has no tight corners. See Fig. 5.

We call the result of trimming an $m_1 \times m_2$ parallelogram board a *trimmed* $m_1 \times m_2$* parallelogram*, which we denote . Similarly, we use  for the result of trimming a triangular board with *m* hexagons on a side. See Fig. 5.

#### Theorem 1.5

The puzzle graphs of the following boards with two holes removed have the strong parity property:

.

Hence flowers, trimmed parallelograms, and trimmed triangles make up an intriguing new family of sliding puzzles that exhibit similar behavior to the classic 15 puzzle. For parallelogram and triangular boards with two holes, the story is a bit more complicated. Tiles can get “stuck” in corners, a behavior that is not seen for square boards. As a result, we obtain many connected components containing non-isolated configurations, as detailed in the following theorem.

#### Theorem 1.6

If the board *B* is

1. A parallelogram , where $m_1,m_2\ge 3$, and $\max \{m_1,m_2\} \ge 4$
2. A triangle  where $$m \ge 5$$

then, the number of connected components of *puz*[*B*(2)] containing non-isolated configurations is given by

- $$\displaystyle 4 \left( {\begin{array}{c}m_1m_2-2\\ 2\end{array}}\right) $$ for a parallelogram,
- $$\displaystyle 12 \left( {\begin{array}{c}m(m+1)/2-2\\ 3\end{array}}\right) $$ for a triangle.

We calculate these God’s numbers using the Breadth-First-Search algorithm that we have run on a specific component of a non-isolated configuration. By symmetry, all components of non-isolated configurations will have the same God’s number.

The rest of the paper is structured as follows. In Sect. 2, we give a proof of Theorem 1.1 and Theorem 1.2. In Sect. 3, we prove a general result that allows us to build boards by gluing smaller boards together in such a way that we preserve the connectivity of the puzzle graph. This allows us to prove Corollary 1.1, which states that the puzzle graph is maximally connected for large-enough triangular and flower-shaped boards with three holes.

Section 4 investigates boards with exactly two holes. Here, parity properties come into play. The section begins with a discussion of *tight corners* of parallelogram and triangular boards, which can be an obstruction to connectivity for the puzzle graph. We then prove Theorem 1.3, which shows that a large class of boards have the *weak* parity property. This establishes Theorem 1.4, which gives the connection number for large-enough parallelogram, triangle, and flower-shaped boards. We adapt our patching method to show that many of the boards in question also have the *strong* parity property, meaning that they are puzzles that exhibit similar behavior as square puzzles (see Theorem 1.5). We end this with Theorem 1.6, which gives the number of connected components containing non-isolated configurations in the puzzle graphs of triangular and puzzle-shaped boards with only two holes. Note that for these boards, the existence of tight corners leads to a large number of components.

Throughout the paper, we make use of inductive arguments, with small boards of a given shape and given number of holes serving as the base case for the induction. We find the God’s number and the number of connected components of the puzzle graphs of these smaller boards computationally, using Python code available in our GitHub repository [9]. Summaries of our computational results, with additional details, are found in Sect. 5.

## First Analysis on Parallelogram-Shaped Hex Boards

First, notice that one may view the board *B*(*h*) as a graph, with vertices and edges corresponding to the corners and sides of hexagonal tiles or holes. It is convenient to consider the planar dual of this graph, which we denote $B^*(h)$.

To construct the dual, we draw a vertex in the center of each hexagon of the board. Two vertices in the *dual graph* are adjacent precisely when the corresponding hexagons share an edge. We may then model the movement of tiles and holes on the original board by assigning tile labels to the corresponding vertices of the dual graph, and letting holes correspond to unlabeled vertices. We may slide a label to an unlabeled vertex precisely when the labeled vertex forms a triangle with two vertices that are unlabeled.

Now, we prove Lemma 1, which will let us, for any board *B*(2) (with certain conditions in its dual graph), assume that the pair of adjacent holes will appear in any location we need. Moreover, the pair of holes will remain adjacent after we perform any sequence of slides.

### Lemma 1

Consider any board *B*(2) such that each triplet of hexagons that are incident in a vertex has two of its hexagons also belonging to another triplet. In the dual graph, this property translates to having any triangle sharing one of its edges with at least another triangle. Assume as well that it has two holes that are (edge) adjacent. Then, ignoring the position of the labels, with a sequence of slides the two adjacent holes can be moved to any position of *B*(2) maintaining their adjacency.

**Fig. 6:**
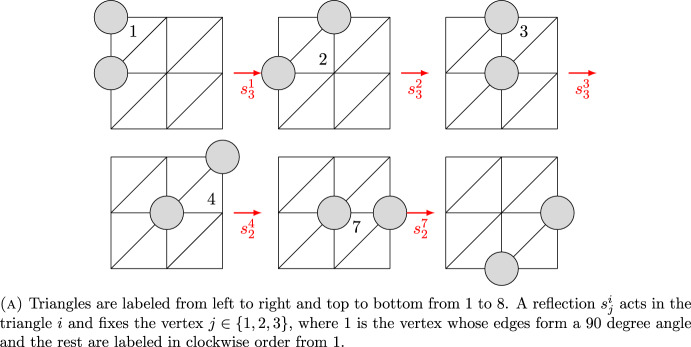
Moving two adjacent holes on a  board as explained in Lemma 1

This Lemma, and also a more general statement assuring that it is possible to move any number of holes from any desired starting configuration to any other desired target position on a board as long as at least two holes stay adjacent, can be proved using the same techniques as in a result by Alpert in [1, Proposition 6]. For completeness, we prove in what follows Lemma 1

### Proof

In the case of a $2 \times 2$ triangle board, the tile can move to any other position, so the 2 adjacent holes can be adjacent in any other position (see Fig. 4). For any other parallelogram, triangular, or flower-shaped board *B*(2), each triplet of adjacent hexagons shares exactly two hexagons with at least one other triplet. Therefore, the dual graph $B^*(2)$ of *B*(2) is a triangulation on the plane in which any triangle shares at least one edge with another triangle. Any adjacent pair of holes in *B*(2) can be represented by an edge $e^i_j \in B^*(2)$, where *i* is a triangle and $j \in \{1,2,3\}$ are each of the possible edges of the triangle *i*. For each pair of triangles that share an edge, we have a relation of the form $e^{i_1}_{j_1}=e^{i_2}_{j_2}$ for some $i_1,i_2,j_1,j_2 \in \mathbb {N}$. Moreover, performing a slide of a tile to a hole is equivalent to applying some reflection $s^i_{j'} \in D_3$, where $D_3$ is the Dihedral group of the triangle, *i* is some triangle, and $j' \in \{1,2,3\}$ is some vertex of the triangle *i* fixed by the reflection. By a sequence of reflections $s^{i_k}_{j'_k} \cdots s^{i_1}_{j'_1}$ centered in different triangles $i_k$, we can take any edge $e^{i_1}_{j_1}$ belonging to the triangle $i_1$ to any other edge $e^{i_k}_{j_k}$ belonging to some triangle $i_k$. We illustrate this process in Fig. 6. $\square$

We observe that all the sequences of boards that we analyze in this paper have the property that in the dual graph, any triangle shares one of its edges with at least another triangle. Next, we prove Theorem 1.1, which states the following:

### Theorem 1.1

For $m_1 =2$ and $m_2 \ge 2$,  has $(m_1m_2-h)!$ connected components containing non-isolated configurations.

### Proof

**Fig. 7:**
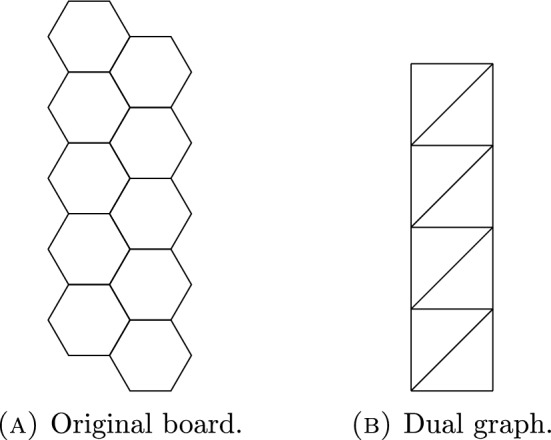
A $2 \times m_2$ hexagonal board with $m_2 = 5$, and its dual graph

We orient our $2 \times m_2$ board so that the hexagonal tiles form columns of length $m_2$ in the vertical direction, with the center of the topmost tile in the left column slightly higher than the center of the topmost tile in the right column. See Fig. 7. $\square$

With these conventions, the dual graph of a $2 \times m_2$ board can be easily deformed to a $2 \times m_2$ grid with rows of length 2 and columns of length $m_2$, and with diagonal edges connecting the bottom-left and top-right of each square. For convenience, we number the rows from top to bottom, starting with row 1.

A configuration of a $2 \times m_2$ board is non-isolated precisely when there are at least two holes in adjacent positions. Let *n* denote the number of tiles. If *n* is even, so that $n = (2m_2-h)= 2k$ for some integer *k*. We can always perform slides on a non-isolated configuration such that the top *k* rows are entirely filled with tiles, and the bottom $m_2 - k$ rows are entirely filled with holes. Similarly, if $n = (2m_2-h)= 2k+1$ for some integer *k*, we can move the tiles so that the top *k* rows are filled with tiles, and there is a tile in the left space of row $k+1$. In either case, we will say a board with tiles and holes positioned in this way is a *home configuration*.

Notice that home configurations are by construction non-isolated as long as $n \le 2m_2 - 2$, which is equivalent to $h \ge 2$. Moreover, every component of  consisting of non-isolated configurations must contain at least one home configuration. The number of home configurations is *n*!, the number of possible permutations of the tiles (keeping the holes fixed). Hence, to prove the proposition, it suffices to show that no two home configurations are in the same connected component of the puzzle graph.

Consider two labels in the dual graph corresponding to a $$2 \times m_2$$ board, say *a* and *b*. We say *a* is $$weakly above $$ *b* if either the row containing *a* is above the row containing *b* or if *a* and *b* are in the same row with *a* on the left. Notice that in a graph corresponding to a home configuration, the label at left in the $$t^{th}$$ row from the top is the unique label with exactly $$2(t-1)$$ labels weakly above it. The label at right in the $$t^{th}$$ row is the unique label with $$2(t-1)+1$$ labels weakly above it.

Consider two home configurations, $C_1$ and $C_2$, which are in the same connected component of the puzzle graph, and let *j* be any tile label which appears in $C_1$ and $C_2$. We claim that the set of labels which are weakly above *j* is the same in the graphs of $C_1$ and $C_2$. In particular, the number of labels which appear weakly above *j* is the same in the graphs of $C_1$ and $C_2$. But this, in turn, shows that *j* must appear in the same position in both configurations, by the argument of the previous paragraph. Hence, all tiles in $C_1$ and $C_2$ appear in the same positions, and $C_1$ and $C_2$ are the same.

We now prove the claim. Suppose *a* is weakly above *b* in the dual graph of a home configuration. It suffices to show that after performing any slide on the graph, *a* will remain weakly above *b*. This is trivial unless the slide moves either *a* or *b*.

**Fig. 8:**
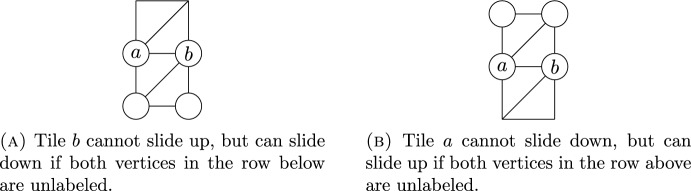
Proof of Case 1: When *a* and *b* are in the same row

Case 1: *a* and *b* are in the same row. Then, *a* must be to the left of *b*. Now, *b* can only slide if there is a triangle in the graph with *b* as one vertex and both other vertices are unlabeled. Since the vertex to the left of *b* is labeled by *a*, the only way *b* can slide is if both vertices in the row directly below *a* and *b* are unlabeled. In this case, *b* may slide down into either spot in that row. But this slide moves *b* into a row below the one containing *a*, so we still have *a* weakly above *b*. See Fig. 8.

Similarly, if we start with *a* and *b* in the same row, and *a* to the left of *b*, then the only case in which *a* can slide is if both spots in the row above *a* and *b* are unlabeled, and *a* slides upward into one of those spots. Again, after such a slide, we still have *a* above *b*.

**Fig. 9:**
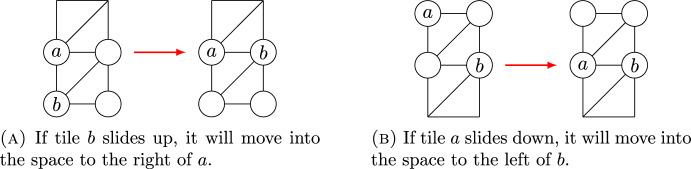
Proof of Case 2: When *a* and *b* are in different rows

Case 2: *a* is in a higher row than *b*. After a slide, each label either remains in the same row or moves to a neighboring row. Hence, it suffices to check the case where *a* is in the row immediately above *b*, and either *a* slides down into the row containing *b* or *b* slides up into the row containing *a*. A tile can only slide upward into a row where one tile is labeled already if the latter labeled tile is on the left, with the unlabeled vertex on its right. Hence, if *b* slides upward into the row containing *a*, then *a* will be to the left of *b* after the slide, as desired. Similarly, for *a* to slide downward into the row containing *b*, the vertex on the left of that row must be unlabeled while the right is labeled by *b*, so again the slide places *a* to the left of *b* as desired. See Fig. 9.

This completes the proof of our claim, and hence of Theorem 1.2. $\square$

We have proved that the board  with $m_2 \ge 2$ generates the same kind of sliding puzzles as the board $R_{\square }(h; 1,m_2)$ for $m_2 \ge 2$. Before studying the puzzle graph of more general parallelogram boards, we first prove a useful lemma that helps us determine when applying a permutation on tiles of a given configuration produces another configuration in the same connected component of the puzzle graph.

### Lemma 2

(Conjugation Lemma) Let *C* be a configuration of a board with *n* tiles, and let $\sigma \in S_n$ be a permutation of the tile labels of *C*. To show that $\sigma \cdot C$ is in the same connected component of the puzzle graph as *C*, it suffices to show that there is a configuration $C_1$ in the same connected component as *C*, such that $\sigma \cdot C_1$ is also in the same connected component as *C*.

### Proof

Suppose we have configurations $C_1$, $\sigma \cdot C_1$ as above in the same connected component as *C*, and $\sigma \in S_n$ is some permutation of the tile labels. Then, we can perform sequences of slides $\tau , \tau ' \in S_{n+h}$ such that $\tau \cdot C = C_1$, and $\tau ' \cdot C_1=\sigma \cdot C_1$. By reversing the slides $\tau$ to take *C* into $C_1$, we can transform $\sigma \cdot C_1$ into $\sigma \cdot C$ as follows

$$\begin{aligned} (\tau ' \tau ^{-1} \tau '^{-1}) \cdot (\sigma \cdot C_1) = \sigma \cdot C, \end{aligned}$$

where the conjugation $\tau ' \tau ^{-1} \tau '^{-1} \in S_{n+h}$ is also a sequence of slides. Therefore, *C* and $\sigma \cdot C$ are in the same connected component of the puzzle graph. $\square$

We now prove Theorem 1.2, which we restate here:

### Theorem 1.2

For $m_1 \ge 3$ and $m_2 \ge 3$, the puzzle graph  is maximally connected.

### Proof

We prove this by simultaneous induction on $m_1$ and $m_2.$ We check the base case, where $m_1 = m_2 = 3$, computationally. See Sect. 5.

Now, suppose the result holds for some $m_1, m_2 \ge 3$. We will show that the result holds for $m_1 + 1, m_2$ and similarly for $m_1, m_2 + 1$. We check the case of $m_1+1,m_2$ here. In this proof, we orient the board so that we have $m_1 + 1$ columns of length $m_2$ running in the vertical direction, with neighboring hexagons in a column sharing a horizontal edge.

Consider any configuration of  where at least two holes are adjacent (so the configuration is non-isolated), and let *a*, *b* be any two tiles. We claim that we can transpose *a* and *b* while leaving the rest of the board fixed. To prove this, we first show that we can perform a sequence of slides that moves *a*, *b* and at least 3 of the holes into some $m_1 \times m_2$ sub-board.

As in Lemma 1 and [1, Proposition 6], Proposition 6, we first slide at least 3 holes into the leftmost column and call the resulting configuration of the board the *ready configuration*. (Note that we may need to move tiles *a* and *b* during this process. We will account for this later.) There are three cases to consider.

Case 1: If *a* and *b* are in the leftmost $m_1$ columns in the ready configuration, then *a*, *b* and three holes are already contained in the sub-board consisting of the first $m_1$ columns, and we are done.

Case 2: If *a* and *b* are both in the rightmost column, slide three holes from the leftmost column to the second column from the left. Then, tiles *a* and *b*, and three holes, are in the sub-board consisting of the rightmost $m_1$ columns.

Case 3: The final case is when *a* is in the rightmost column, but *b* is not. We may then use the base case, applied to the left $m_1$ columns, to perform a sequence of slides that leave three holes and tile *b* in the part of the board excluding the first and last columns. Again, *a*, *b* and three holes are now in the rightmost $m_1$ columns.

Hence, in all three cases we are able to move *a*, *b*, and three holes into some $m_1 \times m_2$ sub-board of the original board. By inductive assumption, we may then switch tiles *a* and *b* without disturbing the rest of the board. By the Conjugation Lemma 2, it follows that starting with any non-isolated configuration, we may switch tiles *a* and *b* while leaving the rest of the board fixed.

Since any permutation of the tiles (keeping the location of the holes fixed) can be achieved with a sequence of transpositions, this proves that we can achieve any permutation of the tiles given a fixed configuration of the holes. By Lemma 1 and [1, Proposition 6], Proposition 6, it is possible to move holes from any configuration with at least two holes adjacent to any other ignoring the tiles labels; this implies that all non-isolated configurations of the board are in the same component of the puzzle graph. $\square$

We offer a different proof of Theorem 1.2 and more general boards in Sect. 3 after we introduce a tool that allows us to extend what we know about the puzzle graph of small boards to the puzzle graph of bigger boards obtained by gluing together copies of the smaller boards.

## Patching Theorem

### Theorem 3.1

(Patching Theorem) Let *B* be a board of any shape with hexagonal tiles. Suppose that *B* may be written as a union of two smaller boards (“patches”) $B_1$ and $B_2$ that satisfy the conditions of Lemma 1 and such that:

- The puzzle graph of the board $B_1(h)$ with $h \ge 3$ holes has one large connected component containing all non-isolated configurations.
- The puzzle graph of the board $B_2(h)$ with $h \ge 3$ holes has one large connected component containing all non-isolated configurations.
- The intersection of $B_1 \cap B_2$ is a connected subset of the board and contains at least $h+1$ hexagons.

Then, for all $h \ge 3$ the puzzle graph of *B*(*h*) has one connected component containing all non-isolated configurations.

### Proof

Let *C* be a non-isolated configuration of the board *B*(*h*), and let *a* and *b* be any two tiles. By the Conjugation Lemma, it suffices to show that there is a configuration $C'$ in the same connected component of the puzzle graph as *C*, such that $(a,b) \cdot C'$ is also in the same connected component.

By Lemma 1 and [1, Proposition 6], we may assume that at least *h* holes are found in the intersection $B_1 \cap B_2$. The puzzle graph of the board $B_1(h)$ with *h* holes has one large connected component containing all non-isolated configurations.

Suppose without loss of generality that *a* is in $B_1 \backslash B_2$ while *b* is in $B_2 \backslash B_1$. Then, we may use the connectedness of the puzzle graph to move tile *b* into $B_1 \cap B_2$ without disturbing any tiles of $B_1 \backslash B_2$, and in such a way that there remain at least *h* holes in $B_1 \cap B_2$. We may then use the connectedness of the puzzle graph of $B_1$ to switch tiles *a* and *b* without disturbing the rest of the board. This proves the lemma. $\square$

**Fig. 10:**
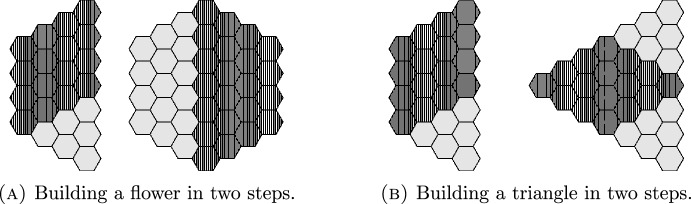
Building boards from parallelogram patches

Corollary 1.1, restated below, is an immediate consequence of the Patching Theorem. We note that Theorem 1.4 is a consequence of the corollary, together with Theorem 1.3.

### Corollary 1.1

For $h \ge 3$, the puzzle graph of  is maximally connected for $m \ge 5$, and the puzzle graph of  is maximally connected for $m \ge 3$.

### Proof

We know that the puzzle graph of  is maximally connected as long as $m_1,m_2 \ge 3$. Our strategy is to build the desired boards out of parallelogram-shaped patches and apply the Patching Theorem.

For the flower-shaped board, suppose we take a  for $n \ge 3$. Then, we may start by overlapping two copies of , so that the overlap is an $n \times n$ equilateral triangle (and hence has more than 4 tiles). This creates a trapezoidal patch. We may then patch together two such trapezoids to form a flower. See Fig. 10.

For a triangular board, the case is even easier. We begin with two parallelograms patched together as above, then add a third parallelogram patch so that all three patches overlap in a central triangle. Again, see Fig. 10. $\square$

## Parity

We now consider flower, triangle, or parallelogram-shaped boards with two holes. Here, parity is a key consideration. In addition, we encounter some obstacles to the connectivity of the puzzle graph, which are not present for square puzzles.

### [Style1 Style2]Definition 8

We say a hexagon is a *tight corner* of a board *B* if the tile has exactly two neighbors, which form a $2 \times 2$ triangle with the tile in question.

Hence, a parallelogram-shaped board has two tight corners, while a triangular board has three. A flower-shaped board has none. Tight corners create some difficulties for boards with only two holes.

### Lemma 1

Consider a board with at least one tight corner. Let *C* be a configuration of the board with exactly two holes and with tile *a* in a given corner. Then every configuration $C'$ in the same connected component of the puzzle graph has either a hole or the tile *a* in the tight corner which is occupied by tile *a* in *C*.

### Proof

If *C* is an isolated configuration, there is nothing to prove. If *C* is non-isolated, then *C* has a single pair of adjacent holes, and so does any $C'$ in the same connected component of the puzzle graph.

Suppose tile *a* is in its original position. To slide tile *a* out of the tight corner, we must move the two holes into the hexagons neighboring the corner. We may then slide tile *a* into either hole, leaving a hole in the corner. Once this happens, however, the only tile capable of sliding is *a*. We may either slide tile *a* back to its original position, or slide *a* into the other hole–which leaves a hole in the tight corner.

Hence, if *a* is in its original tight corner sliding *a* out of the tight corner leaves a hole in its place. If *a* is no longer in its corner, all possible slides result in the corner being occupied by a hole or *a* itself. This proves the lemma. $\square$

### Remark 2

We note that a trimmed parallelogram board may be obtained by taking a parallelogram-shaped board, and removing the two tight corners. Also, a trimmed triangle may be obtained by taking a board shaped like an equilateral triangle, and removing the three tight corners. A trimmed parallelogram  has no tight corners for $m_1,m_2 \ge 3$, and a trimmed triangle  has no tight corners for $m \ge 4$.

### Lemma 3

Let *B* be the board , or the parallelogram-shaped  where $m_1,m_2 \ge 3$. Let $B'$ be the trimmed board obtained by removing the tight corners of *B*, and suppose the puzzle graph of the board $B'$ with two holes has *c* connected components containing non-isolated configurations.

Then, the number of connected components of the puzzle graph of *B* with two holes and containing non-isolated configurations is

- $$\displaystyle 2c \left( {\begin{array}{c}m_1m_2-2\\ 2\end{array}}\right) $$ for a parallelogram,
- $$\displaystyle 6c \left( {\begin{array}{c}m(m+1)/2-2\\ 3\end{array}}\right) $$ for a triangle.

### Proof

Let $C_1$ be a non-isolated configurations of the board *B* with no holes in tight corners, and let $C_2$ be another such configuration in the same component of the puzzle graph. Let $C_1'$ be the configuration of $B'$ induced by $C_1$. As in the proof of Lemma 1, we may slide a tile out of a tight corner of the board *B*; however, this tile must be returned to its original position without disturbing the rest of the board before any slides involving other tiles can take place. Hence, we may assume that the sequence of slides transforming $C_1$ into $C_2$ involves no slides into or out of tight corners. The possibilities for $C_2$ are then in bijection with the non-isolated configurations of $B'$ with two holes removed, which are in the same connected component of the puzzle graph of $B'(2)$ as $C_1'.$

**Fig. 11:**
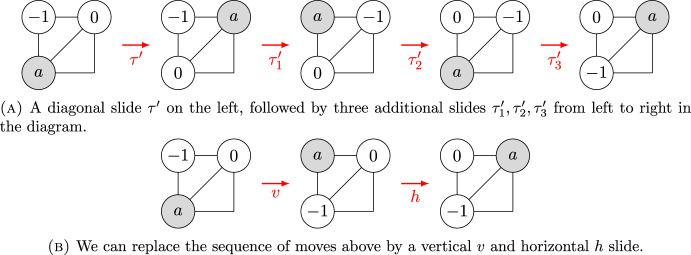
Dealing with diagonal slides in the proof of Lemma 4

Let *t* be the total number of tiles on the board *B*. It follows that configurations with no holes in their tight corners may be partitioned into $$\displaystyle c \cdot k! \left( {\begin{array}{c}t\\ k\end{array}}\right) $$ connected components, where *k* is the *number of tight corners* of *B*. We have $$\displaystyle k! \left( {\begin{array}{c}t\\ k\end{array}}\right) $$ options for the placement of tiles in the tight corners, and we obtain *c* distinct connected components for each choice of tight corner tiles. Since every non-isolated configuration is in the same connected component as a configuration with no holes in tight corners, we have in fact given a complete count of connected components containing non-isolated configurations, and the lemma has been proved. $\square$

Having addressed the problem of tiles getting stuck in tight corners, we now turn to a more fundamental obstacle to the connectivity of the puzzle graph: parity of permutations on tile labels.

### [Style1 Style2]Definition 9

We define an *augmented configuration* of  to be a configuration of  where the two holes have been labeled 0 and $-1$. The rules for sliding tiles remain the same as when the holes were unlabeled.

Working on an augmented configuration of  will help us track the holes’ position. Before we prove Theorem 1.3, which states that parallelogram boards with two holes have the weak parity property, we shall prove the next lemma.

### Lemma 4

Let $C_1$ and $C_2$ be two configurations on the same component of the puzzle graph *puzz*[*B*(*h*)]. There exists a finite sequence of horizontal or vertical slides such that the final configuration of the labeled tiles is $C_2$.

### Proof

Since $C_1$ and $C_2$ are in the same component, there exists a finite sequence of slides such that $(\tau _n \cdots \tau _1) \cdot C_1 = C_2$, where the $\tau _i \in S_{n+h}$. We claim that the $\tau _i$ can be considered a sequence of horizontal and vertical slides.we Indeed, if $\tau '$ is some diagonal slide, after applying $\tau '$ we can perform a sequence of three additional slides $\tau '_3 \tau '_2 \tau '_1$ such that $\tau '_3 \tau '_2 \tau '_1 \tau '$ is a vertical slide followed by a horizontal slide. In Fig. 11, we explain this sequence of slides for the case of an upper-right diagonal move, but if the move is in the lower-left direction, the process is analogous. $\square$

### Lemma 5

By restricting to horizontal or vertical slides on the augmented configuration of , the lexicographic order of the holes labeled by $-1$ and 0 remains unchanged.

### Proof

Consider the dual graph of  and let $p_{-1}=(x_{-1}, y_{-1})$, and $p_{0}=(x_{0}, y_{0})$ be the coordinates of the holes labeled by $-1$ and 0 respectively, with the origin on the lower-left position of the board. Without loss of generality, suppose $p_{-1} \prec p_{0}$ with the lexicographic order, that is $x_{-1}<x_0$ or $x_{-1}=x_0$ and $y_{-1}<y_{0}$. Next, we analyze the possible cases:

1. If $x_{-1}<x_0$, then it can happen that $y_{-1}=y_0$ or $y_{-1}<y_0$ (note that $y_{-1}>y_0$ cannot hold because the holes would be separated). We consider the following sub-cases:
  In the first sub-case, tile *a* can only move up to replace hole $-1$ and still $x_{-1}<x_0$, or *a* could slide down to replace hole 0 and still $x_{-1}<x_0$.
  In the second sub-case, tile *a* can move left, replacing hole $-1$ leaving $x_{-1}=x_0$ and $y_{-1}<y_0$ or *a* can go up, replacing hole 0 leaving $x_{-1}<x_0$. Similarly a tile *a* can move right, replacing hole 0 leaving $x_{-1}=x_0$ and $y_{-1}<y_0$ or *a* can go down, replacing hole $-1$ leaving $x_{-1}<x_0$.
2. If $x_{-1}=x_0$ and $y_{-1}<y_0$, then only horizontal moves can be performed. If tile *a* moves right, replacing hole $-1$, then $x_{-1}<x_0$ holds, and if tile *a* moves left, replacing hole 0, then $x_{-1}<x_0$ holds.

Therefore, lexicographic order is preserved when applying horizontal or vertical slides. $\square$

### Theorem 1.3

The board  has the weak parity property for $m_1, m_2 \ge 3.$

### Proof

We work with the corresponding dual graph of the board . That is, with the grid that has one vertex per each hexagonal cell on  and that has one edge connecting two of these vertices if the corresponding hexagons share an edge. See Fig. 7 for a small example. Let $\bar{C}$ be an augmented configuration obtained by labeling the holes of *C* with $-1$ and 0.

For any augmented configuration $\bar{C'}$ whose underlying configuration is in the same component of  as *C*, we define the *augmented parity* of $\bar{C'}$ as follows. We ignore the diagonals of the dual graph and consider only the vertical and horizontal edges. Let $p_1$ be the parity (zero for even, one for odd) of the taxicab distance from the hole labeled $-1$ to the lower-left vertex of the grid. Let $p_2$ be the parity of the taxicab distance of the hole labeled 0 to the lower-left vertex. Finally, let $p_3$ be the parity of the permutation $\tau \in S_{n+2}$ of *all* positions on the board (both tiles and labeled holes) needed to transform $\bar{C}$ into $\bar{C}'$. We define the augmented parity of $\bar{C}'$ as the parity of the sum $p_1+p_2+p_3$.

We now claim that we can transform *C* into $C'$ using a sequence of moves that preserve the augmented parity. First, consider the case where we slide a tile vertically or horizontally. A single slide transposes a hole and a tile, which changes the parity $p_3$ of the overall permutation of positions on the board. In addition, with each vertical or horizontal slide, exactly one of the holes moves a distance of one in the horizontal or vertical direction, while the other hole does not move at all. So exactly one of $p_1$ or $p_2$ changes. Hence, each vertical or horizontal slide changes exactly one of either $p_1$ or $p_2$, while $p_3$ always changes. It follows that such a slide does not change the augmented parity of the augmented configuration.

This is not the case with diagonal slides. Note that when a tile slides diagonally, the parity of its taxicab distance from a given point will not change, as a diagonal slide is equivalent to moving one position in the horizontal direction and one position in the vertical direction. Hence, a diagonal slide changes the augmented parity because $p_1$ and $p_2$ remain unchanged while $p_3$ changes. Fortunately, by Lemma 4 we can replace any diagonal slide by a sequence of vertical or horizontal slides, which won’t change the augmented parity.

It remains to show how the augmented parity relates to the parity of the permutation of the tile labels. First note that Lemma 5 guarantees that after performing a sequence of horizontal or vertical slides and returning the holes as in the initial configuration, the lexicographic order of their labels $-1$ and 0 is preserved. Therefore, the hole labels $-1$ and 0 are not permuted. Note that there are two possible initial configurations of the labeled holes:

1. Suppose $p_1+p_2$ is initially even. Since the identity permutation is even, then $p_3=0$. Therefore, the augmented parity $p_1+p_2+p_3$ is initially even. After performing a sequence of horizontal and vertical slides and returning the holes to their initial positions, the augmented parity does not change. Therefore, at the final configuration $p_1+p_2+p_3$ will be even. So, $p_3$ must be even in the final configuration.
2. Suppose $p_1+p_2$ is initially odd. Since the identity permutation is even, then $p_3=0$. Therefore, the augmented parity $p_1+p_2+p_3$ is initially odd. After performing a sequence of horizontal and vertical slides and returning the holes to their initial positions, the augmented parity does not change; therefore, at the final configuration $p_1+p_2+p_3$ will be odd. So, $p_3$ must be even in the final configuration.

Finally, note that if the permutation $\tau \in S_{n+2}$ of the tile labels and holes is even and the holes are not permuted, then the permutation $\sigma \in S_n$ of the tile labels must be even. $\square$

We can now prove Corollary 1.2 that we restate below.

### Corollary 1.2

Any board with exactly two holes that is a sub-board of a parallelogram board has the weak parity property. Hence, boards of any shape that we explore in this paper with exactly two holes have the weak parity property.

### Proof

Let *B* be a board which can be embedded in a larger parallelogram-shaped board *P*. Suppose we can apply a sequence of slides to the board *B*(2) of shape *B* with exactly two holes and achieve an odd permutation of the tiles. Viewing *B* as a sub-board of *P*, we have a sequence of slides which produces an odd permutation of the tiles of *P*(2), contradicting Theorem 1.3. $\square$

Now, using also Corollary 1.1, we can finish the proof of Theorem 1.4 which states:

### Theorem 1.4

The connection number is 3 for the following boards:  with $m_1,m_2 \ge 3$,  with $m \ge 5$, and  for $m \ge 3.$

### Proof

By Theorem 1.2 and Corollary 1.1, the puzzle graphs of large-enough parallelogram, triangle, or flower-shaped boards are maximally connected if they have three or more holes. By Theorem 1.3, the puzzle graphs of these boards are not maximally connected if they only have two holes. $\square$

Before giving a variation on the Patching Theorem for boards with exactly two holes, which will allow us to use patching to prove the strong parity property, we shall prove the following lemma.

### Theorem 4.1

(Strong Parity Property via Patching) Suppose *B* is a board which can be written as a union of two smaller boards, or “patches”, $B_1$ and $B_2,$ such that all of the following hold:

1. $B_1$ and $B_2$ each contain at least five hexagons.
2. $B_1 \cap B_2$ contains at least four hexagons, at least two of which are adjacent.
3. When we consider $B_1$ and $B_2$ separately, each one has, with exactly two holes, the strong parity property.

Then if *B* with exactly two holes has the weak parity, it has the strong parity property as well.

### Proof

Recall that the alternating group of even permutations is generated by 3-cycles of the form (*a*, *b*, *c*). Let *C* be a non-isolated configuration of the board *B*, and let *a*, *b*, *c* be any three tiles. By the Conjugation Lemma, it suffices to show that there is a configuration $C'$ in the same connected component of the puzzle graph as *C*, such that $(a,b,c) \cdot C'$ is also in the same connected component.

We may assume that both holes are in the intersection $B_1 \cap B_2$. If *a*, *b*, and *c* are in the same patch (say, $B_1$), then we may use the assumptions about the puzzle graph of $B_1$ to apply the 3-cycle (*a*, *b*, *c*), without disturbing the rest of the board.

Suppose without loss of generality that $a,b \in B_1$ while *c* is in $B_2 \backslash B_1$. We may assume further that $a \in B_1 \backslash B_2$, or else we would be in the first case. Note, however, that we may have $b \in B_1 \cap B_2$. We use the assumption on $B_2$ to move tile *c* into $B_1 \cap B_2$, without disturbing any tiles of $B_1 \backslash B_2$, and in such a way that the two holes remain in $B_1 \cap B_2$, and *b* remains in $B_1 \cap B_2$ if needed. (Note that we can do this with an even permutation of tiles, since $B_2$ has at least three labeled tiles.) We may then use the parity property of $B_1$ to apply the permutation (*a*, *b*, *c*). This proves the lemma. $\square$

We provide now the proof of Theorem 1.5.

### Theorem 1.5

The puzzle graphs of the following boards with two holes removed have the strong parity property:

.

### Proof

We begin with the case . We check this computationally—see our discussion in Sect. 5. It follows by induction that the result holds for all larger trimmed parallelograms, as we may cover any such board with two smaller trimmed parallelogram patches, whose intersection covers either all but the first and last rows of the board or all but the first and last columns, and then apply Theorem 4.1.

**Fig. 12:**
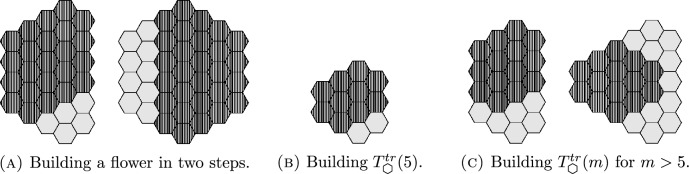
Building boards from trimmed parallelogram patches

The flower  may be constructed by joining two copies of , as shown in Fig. 12, and then joining two of the resulting patches to form a flower. See Fig. 12. Note that this is analogous to the way we built up flower-shaped boards for parallelogram patches in Fig. 10.

For a trimmed triangle, the smallest case can easily be constructed from two trimmed $3 \times 4$ parallelogram patches. Larger trimmed triangles may be constructed by successively joining three trimmed parallelogram patches, as in Fig. 12. $\square$

We can now easily prove Theorem 1.6, which we restate below.

### Theorem 1.6

If the board *B* is

1. A parallelogram , where $m_1,m_2\ge 3$, and $\max \{m_1,m_2\} \ge 4$
2. A triangle  where $$m \ge 5$$

then, the number of connected components of *puz*[*B*(2)] containing non-isolated configurations is given by

- $$\displaystyle 4 \left( {\begin{array}{c}m_1m_2-2\\ 2\end{array}}\right) $$ for a parallelogram,
- $$\displaystyle 12 \left( {\begin{array}{c}m(m+1)/2-2\\ 3\end{array}}\right) $$ for a triangle.

### Proof

Applying Theorem 1.5, we can use $c = 2$ in Lemma 3. $\square$

## Computational Results for Smaller Boards

We now check computationally the smaller triangular, parallelogram, trimmed parallelogram, and flower-shaped boards. We note that it is not necessary to compute the entire puzzle graph to determine the number of connected components. Indeed, computing a single connected component is enough, as we explain below. The Python code for computing a list of all configurations in a sample connected component, as well as the complete output files from our computations, can be found in our GitHub repository [9].

### Proposition 1

Let *B*(*h*) be a board satisfying the conditions of Lemma [1, Proposition 6] with *h* holes, and let *S*, $S'$ be two connected components of *puz*[*B*(*h*)]. Then, there is a graph isomorphism from *S* to $S'$ which preserves the locations of the holes in a configuration.

### Proof

Consider two configurations *C* and $C'$ of *B*(*h*) which have holes in precisely the same positions, but which do not lie in the same component of *puz*[*B*(*h*)]. This means that there is a permutation of the tile labels $\sigma \in S_n$ such that $\sigma \cdot C = C'$. Note that if $\tau$ is any sequence of slides, then permuting the tile labels commutes with sliding tiles on the board, that is $\tau \sigma = \sigma \tau$. Hence, the permutation of labels $\sigma$ induces a graph isomorphism between the connected component of *puz*[*B*(*h*)] containing *C* and the component containing $C'$. Moreover, this isomorphism preserves the positions of the holes in a configuration. $\square$

### Corollary 5.1

Let *t* be the number of tiles of *B*(*h*). The number of connected components of *puz*[*B*(*h*)] containing non-isolated configurations is *t*! divided by the number of configurations in any such connected component of *puz*[*B*(*h*)] which have their holes in a specified set of positions.

### Proof

Suppose we choose a set of positions for the holes of *B*(*h*) which yields a non-isolated configuration and call any configuration of *B*(*h*) with holes in the specified positions a *home configuration*. Let *t* be the number of tiles on the board *B*(*h*). The total number of home configurations of *B*(*h*) is hence *t*!, the number of possible permutations of the tile labels. By the argument in the preceding paragraph, each connected component *puz*[*B*(*h*)] containing non-isolated configurations contains the same number of these home configurations. Thus, the number of such connected components is *t*! divided by the number of home configurations found in a given component. $\square$

### The Board 

In Table 1, we give the computational results that we have obtained for the flower-shaped board , which has six hexagonal tiles arranged in a ring around a central tile. We include here the total number of configurations in a connected component of the puzzle graph in order to convey the difficulty level of the corresponding sliding puzzles.

**Table 1:** Results for

Holes	Components	Component size
2	24	60
3	6	132
4	1	210

### Small Triangular Boards

In Table 2 we show the results for the triangular board , where $m=2,3,4$. For each *m*, we check values of *h* starting at 2 until we reach the connected number of . Again, components here refer to components of the puzzle graph containing non-isolated configurations.

**Table 2:** Results for

*m*	Holes	Components	Component size
2	2	1	3
3	2	24	9
3	3	6	19
3	4	1	30
4	2	8064	90
4	3	1	498960

The results for $m=2$ can be found easily by inspection. For $m=3$, we use Python to compute a single component, and apply Corollary 5.1. We use this same approach for the case $m=4, h=3$.

For the case $m=4$, $h=2$, we instead applied Lemma 3, together with our results on  to give the number of connected components. To obtain the number of configurations in a component, note that for  we have 60 configurations per component, with 5 configurations for each possible position of the two adjacent holes. This gives 60 configurations of the triangular board, where no holes are found in the tight corners. For each corner, there are 5 possible configurations of the board where the two holes are adjacent to the given corner, and we may then slide a tile from the tight corner into either of the holes. Hence, we have 10 configurations with a hole in the given corner, giving 30 more configurations, for a total of 90. This counting method was less time consuming than running the needed computations on the author’s laptop.

### Parallelograms, Trimmed Parallelograms, and Parity

Computationally, we find that  has a puzzle graph that is maximally connected, while  has a puzzle graph with exactly two connected components containing non-isolated configurations. By the proposition below, it follows that  has the strong parity property.

#### Proposition 2

If *B*(*h*) has the weak parity property, and *puz*[*B*(*h*)] has exactly two connected components containing non-isolated configurations, then *B*(*h*) has the strong parity property.

#### Proof

Note that the number of even permutations of *t* tiles is *t*!/2. Hence, for each of the two components of *puz*[*B*(*h*)] containing non-isolated configurations, the number of configurations with holes in a specified set of positions is equal to the number of even (respectively odd) permutations of the tile labels. It follows that all possible even permutations of the tile labels must be found in one large connected component, while all possible odd permutations must be found in the other. $\square$

### God’s Number for Small Boards

In addition to finding the number of configurations in a sample connected component of the puzzle graph, our Python code allows us to find bounds on God’s number for small puzzles. Our code uses a Breadth-First Search (BFS) algorithm. We begin with a starting configuration, and iterate as follows. At the $n^{th}$ iteration, we start with a list of configurations at distance less than or equal to *n* from the starting configuration. We then find any neighbors of the configurations at distance *n* from the start that are not already on the list. These are precisely the configurations that are at distance $n+1$ from the original configuration. We add these new configurations to our list and repeat the process. The algorithm terminates when it no finds any new configurations, meaning that we have reached the farthest point in the puzzle graph from our original configuration. Hence, the algorithm computes a spanning tree for the component of *puz*[*B*(*h*)] containing our starting configuration, with the starting configuration as a root.

We note that God’s number is the maximum depth of a tree computed using the BFS algorithm, over all possible starting configurations. By symmetry, it is enough to check configurations with all possible starting positions of the holes, as the trees for two configurations with holes in the same starting locations will be isomorphic.

#### Proposition 3

Let *d* be the depth of the tree computed by our BFS algorithm when building a component of the puzzle graph *puz*[*B*(*h*)], starting from configuration *C*. Then, God’s number for the board *B*(*h*) is bounded below by *d*, and above by 2*d*

#### Proof

Since *d* is the distance from *C* to some configuration $C'$, *d* is certainly a lower bound on God’s number.

We can find a path between any two configurations *D*, $D'$ by first finding a path from *D* to *C* in the spanning tree, and then a path from *C* to $C'$. Since every configuration is a distance of at most *d* from *C*, concatenating these two paths gives a path of length at most 2*d* from *D* to $D'$. The result follows, since all components of *puzz*[*B*(*h*)] containing non-isolated configurations are isomorphic. $\square$

Using our BFS algorithm and the arguments given above, we found bounds for the God’s numbers of some of the boards. We resume these results in Table 3. We conjecture that, in many cases, the depth of a single tree is in fact God’s number for the board overall, but we have not verified this computationally.

**Table 3:** Bounds on God’s number for some small boards

Board	Holes	Lower bound	Upper bound
	2	16	32
	3	12	24
	4	8	16
	2	3	6
	3	4	8
	4	6	12
	2	17	34
	3	56	112
	3	51	102
	2	78	156

## Final Remarks and Open problems

In our GitHub repository [9], we provide 3D models for the small hexagonal boards that we have studied in this paper. These have been kindly developed by Henry Segerman and can be used for 3D printing and playing with some of the hexagonal sliding puzzles that we have studied here.

Recently [1], the asymptotic growth of God’s number of $puz[R_{\square }(h;m)]$ with $h \ge 2$, and  with $h \ge 6$ and $m=k^2$ for $k\ge 1$ has been determined up to a constant factor. In these cases, the puzzle graph is maximally connected. Finding God’s number for the family of boards that we have studied here remains an open problem. Here, we have given bounds for the God’s numbers for some small hexagonal boards. It is possible to use the algorithms that we have implemented to find the exact values of the God’s number for these sliding puzzles by performing an exhaustive search.

For generating the puzzle graph, labeled tiles are only allowed to be on positions determined by the vertices of the dual graph of the underlying tessellation. A higher dimensional topological space that contains the puzzle graph is generated by allowing the tiles to move continuously on the board as long as two tiles do not overlap. This topological space has been defined and studied for square sliding puzzles on boards with a rectangular shape; it is called the configuration space of hard squares on a rectangle [5, 6]. Recently [6], it has been proven that the configuration space of the 15 Puzzle deformation retracts to the puzzle graph of $R_{\square }(1;4,4)$. More generally, in this paper it was proven that for the configuration space of rectangular-shaped boards $R_{\square }(1;m_1,m_2)$, deformation retracts to a one-dimensional subspace that is homeomorphic to $puz[R_{\square }(1;m_1,m_2)]$. This relationship between the puzzle graph and the configuration space of $R_{\square }(h;m_1,m_2)$ no longer holds true for $h\ge 2$, which is when maximal connectivity happens. In the case of the hexagonal sliding puzzles that we have studied here, it could be the case that before maximal connectivity the same topological relationship will hold between the puzzle graph and the configuration space.

## Data Availability

The authors declare that the data presented here and the code used to generate it are available in [9].
